# Development of an Ultrasonic Airflow Measurement Device for Ducted Air

**DOI:** 10.3390/s150510705

**Published:** 2015-05-06

**Authors:** Andrew B. Raine, Nauman Aslam, Christopher P. Underwood, Sean Danaher

**Affiliations:** Faculty of Engineering and Environment, Northumbria University, Newcastle upon Tyne NE18ST, UK; E-Mails: andrew.raine@northumbria.ac.uk (A.B.R.); chris.underwood@northumbria.ac.uk (C.P.U.); sean.danaher@northumbria.ac.uk (S.D.)

**Keywords:** acoustics, airflow rate, ultrasonic flowmeter, ventilation air flow measurement, differential transit time method, time difference method, ultrasonic transducer damping, ultrasonic receiver amplifier

## Abstract

In this study, an in-duct ultrasonic airflow measurement device has been designed, developed and tested. The airflow measurement results for a small range of airflow velocities and temperatures show that the accuracy was better than 3.5% root mean square (RMS) when it was tested within a round or square duct compared to the in-line Venturi tube airflow meter used for reference. This proof of concept device has provided evidence that with further development it could be a low-cost alternative to pressure differential devices such as the orifice plate airflow meter for monitoring energy efficiency performance and reliability of ventilation systems. The design uses a number of techniques and design choices to provide solutions to lower the implementation cost of the device compared to traditional airflow meters. The design choices that were found to work well are the single sided transducer arrangement for a “V” shaped reflective path and the use of square wave transmitter pulses ending with the necessary 180° phase changed pulse train to suppress transducer ringing. The device is also designed so that it does not have to rely on high-speed analogue to digital converters (ADC) and intensive digital signal processing, so could be implemented using voltage comparators and low-cost microcontrollers.

## 1. Introduction

Currently heating, ventilation and air conditioning (HVAC) systems are difficult and costly to monitor for energy efficiency performance and reliability. A typical duct airflow monitoring device uses a pressure differential method to determine the airflow. According to Yu *et al.* [1] they are fragile, expensive and create an additional pressure loss, and their sensitivity is also reduced with the reduction in airflow velocity. As buildings evolve, they will require higher levels of insulation and air tightness which will require ventilation systems to provide the minimum number of air changes and reduced energy usage by recovering heat from the air before it is expelled. This will necessitate the need for monitoring of the operating performance of these systems so that air quality or building energy efficiency is not detrimentally affected [2,3]. The monitoring of airflow rates can indicate problems in the design, installation and operation of a HVAC system [2,3,4].

One of the possible alternatives, listed on pages 36.15 and 36.21 of [5], to using pressure differential type devices such as Pitot tube/arrays, orifice plates and Venturis is to use an ultrasonic flow rate sensor which, according to Lynnworth and Liu [6], have been around for at least 60 years [7]. Ultrasonic flow sensors are commonly used to measure pipeline liquid flow in industrial applications but they are not as common for gas flow. There are commercially available systems for use in monitoring industrial processes such as exhaust gases [8] and automotive test bed air intakes [9] but the historically high cost [10,11] has restricted their application uses in HVAC systems.

A duct airflow measurement device was proposed by Bragg and Lynnworth [12] from Panametrics Inc. (Waltham, MA, USA) at a conference in 1994 which used a single port solution consisting of two transducers mounted using O-rings to reduce crosstalk on a single circular flange. A reflective path was used and it was suggested that optional sensors could be combined with this unit such as pressure, temperature and relative humidity. In 2002 a similar but more developed device was described by Rabalais and Sims [13] but was only available on a case-by-case basis as they were still classed as experimental devices. In 1994 a patent was filed by Strauss *et al.* [14], which described a solution for measuring HVAC air velocity by using a contra-propagating pulsed phase method.

The measurement of low airflow velocities in natural ventilation systems has created an interest in developing an ultrasonic airflow velocity measurement device. This is because of the linear response to flow velocity change that these devices have, so their sensitivity does not degrade with low airflow velocity as opposed to what happens with pressure differential airflow measurement devices. In a study by Olmos [10] an ultrasonic airflow measurement device for measuring airflow and temperature within solar chimneys was developed. This incorporated a contra-propagating pulse phase method with time of flight tracking similar to a method used by the same author in a previous study for an ultrasonic tank level meter [15]. In another study by van Buggenhout *et al.* [11] on natural ventilation air flow measurement, a device was created which could use between 1 and 16 transducer pairs fitted in a circular duct to measure the turbulent airflow to an accuracy of 9% for all 16 pairs and 24% for a single pair. 

The objectives of this study were to develop a proof of concept for a practical airflow measurement device for 0 to 10 m/s low velocity ducted air with an accuracy similar to typical existing air velocity measurement devices [5] (p. 36.15), of between 2% and 5%, and with the potential to be developed at lower cost than the currently available ultrasonic in-duct airflow measurement or pressure differential devices. The fully developed device would be expected to operate with in-duct airflow temperatures from −30 °C to 60 °C together with duct sizes of height ranging from 100 mm to 1000 mm over the full humidity range. To aid this process, an ultrasonic airflow measurement development system was designed and constructed which involved a significant amount of hardware and software integration. The research and development work involved testing of various methods and techniques to discover a system which would be less costly to implement with an acceptable quality of measurement. 

## 2. Theory

**Figure 1:**
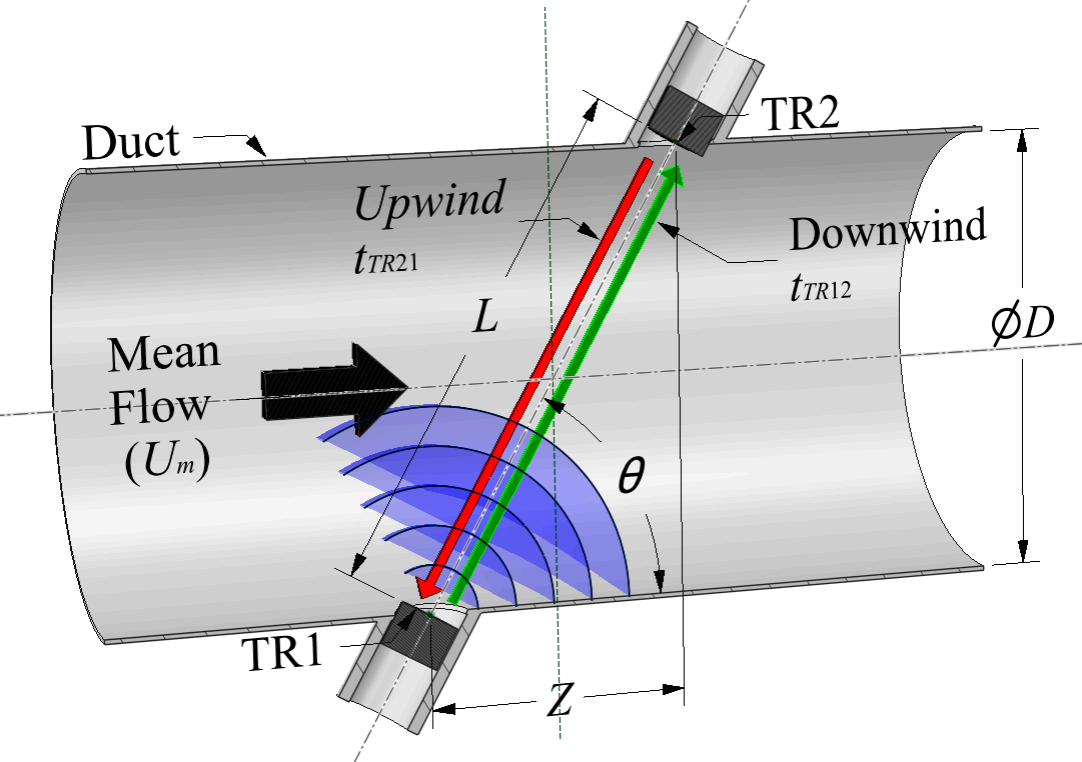
Single path acoustic transit time flow meter in a circular duct.

The transit time acoustic flow meter, as shown in Figure 1, works on the principle that sound waves will propagate faster in the direction of the flow than against it [16,17]. The following section now explains how to predict transit times and calculate the flow velocity. Two different methods of calculating the flow velocity are described. The first method uses the absolute transit times and the other uses the direct measurement of the transit time difference plus airflow temperature.

The approximate velocity of the speed of sound *c*, in still dry air of temperature *t* in degrees Celsius is given by the following Equation (1) [18]:

(1) $$c\approx 331.45\sqrt{1+\frac{t}{273}}\;\text{m/s}$$

The single path acoustic transit time flow meter geometric parameter values are given by the following Equations (2) and (3):

(2) $$L=\sqrt{D^{2}+Z^{2}}$$

(3) $$\theta =\tan^{-1}\frac{D}{Z}$$

Hence the transit time can be predicted by Equations (1)–(3) and the following Equation (4):

(4) $$t_{TR\;=}\;\frac{L}{c\pm U_{m}\cos\theta }$$

The mean airflow velocity *U_TTM_* across the acoustic path can be calculated using Equation (5) [19] without the need to know the speed of sound:

(5) $$U_{TTM}=\;\frac{L}{2\cos\theta }\left(\frac{1}{t_{TR12\;}}-\frac{1}{t_{TR21\;}}\right)$$

To reduce errors caused by noise and fluctuating signal levels, the absolute transit time is usually measured by using the cross-correlation [20] digital signal processing (DSP) method [21]. This compares the digital representation of the transmitted and received waveforms to calculate the delay between them to reduce errors caused by noise and fluctuating signal levels. Threshold crossing techniques can be used as well [22]. In this study, a phase shift or differential transit time method similar to that described by Han *et al.* [19] and de Cicco *et al.* [23] is used to reduce the cost of implementation for this application. In the differential transit time method, the estimated transit time is found by using a two stage calculation. In the first stage, the zero flow transit time is calculated with Equation (6). In this equation the acoustic path length in Equation (2) is divided by the speed of sound in Equation (1), which is derived from the airflow temperature:

(6) $$t_{U0}=\;\frac{L}{c}$$

In the second stage, half the actual measured differential transit time is added or subtracted from the zero flow transit time depending on the airflow direction, which is represented in Equation (7) below:

(7) $$t_{TR\;}=t_{U0}\pm \frac{\Delta t_{TR}}{2}$$

The airflow velocity can then be calculated by using the inverse transit time difference (ITTD) [24] formula presented in Equation (5).

## 3. Design

### 3.1. HVAC Ultrasonic Duct Airflow Measurement Development System

**Figure 2:**
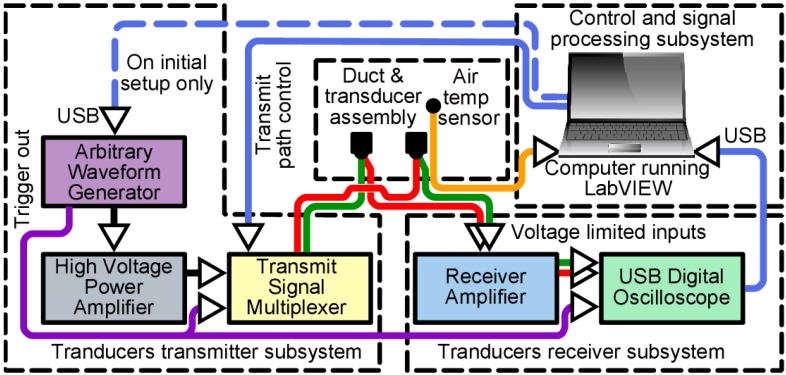
Schematic of the ultrasonic duct airflow measuring device development system.

To develop the ultrasonic duct airflow measurement device a system was produced which had the flexibility to test various measurement techniques. An overview of the development system is shown in Figure 2 which is divided into four subsystems. These subsystems are individually discussed in the next four Section 3.2, Section 3.3, Section 3.4 and Section 3.5, together with the final configuration method used for testing. The rest of this section contains a general overview of the system operation and equipment used.

A computer controlled arbitrary waveform generator (AWG) was used so that any type of transmit drive signal could be produced and manipulated quickly by the extensively modified LabVIEW*^®^* 10 (National Instruments, Austin, TX, USA) instrument driver software. The output of the AWG was fed into a PA95 (Apex Microtechnology, Tucson, AZ, USA) high voltage power amplifier capable of driving the ultrasonic transducer to its maximum voltage of approximately 100 V peak to peak. This was then switched through two LH1500AT (Vishay Intertechnology, Malvern, PA, USA) solid-state relays (SSR) within the high voltage multiplexer specifically designed for the task which could switch a single input between at least two outputs. The multiplexer was also controlled by the computer through a PicoLog*^®^* 1012 (Pico Technology, St Neots, UK) universal serial bus (USB) data acquisition device. The transmit signals were then connected, depending on the multiplexer state, to one of two 400EP14D (Pro-Wave Electronic Corp., New Taipei City, Taiwan) 40 kHz enclosed type piezoelectric transducers which would transmit an ultrasonic signal through the duct to a receiving transducer. The received signal would be then amplified after passing through a diode voltage limiter input circuit to a multistage operational amplifier to boost the signal voltage gain to >1000. This was then digitized by a USB oscilloscope to be processed by the computer running the LabVIEW*^®^* control and signal processing software. A Grant type U thermistor probe was used to measure the in-duct air temperature and was monitored via a Squirrel^®^ SQ2020 Series Data Logger (Grant Instruments, Shepreth, UK).

### 3.2. Transducer Configuration

A single reflective path ultrasonic flow meter design was chosen as the preferred solution. The transducers are mounted on the same side so that they can be constructed as a single assembly which is fitted to the duct wall. This should reduce the overall cost as it simplifies installation and reduces the number of cable assemblies required.

The single reflective “V” shaped path geometric parameter values are given by the following Equations (8) and (9):

(8) $$L=\sqrt{2D^{2}+Z^{2}}$$

(9) $$\theta =\tan^{-1}\frac{2D}{Z}$$

**Figure 3:**
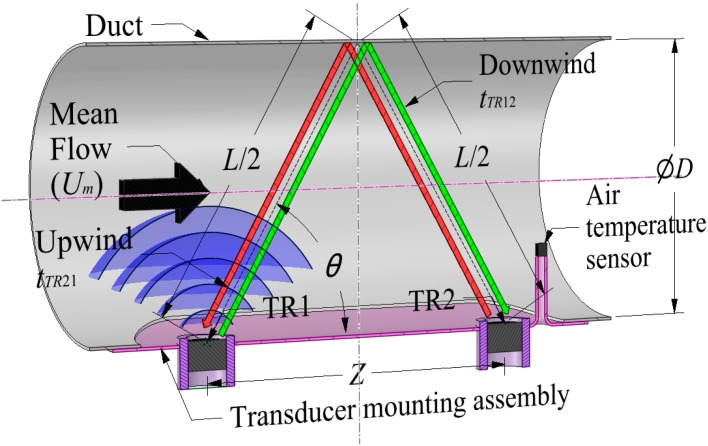
A reflective “V” shape single path acoustic differential transit time flow meter in a cylindrical duct.

Figure 3 shows a similar device to Figure 1 but using a reflective “V” shaped path with the transducers mounted perpendicular to the duct wall.

To explore the sensitivity of airflow measurements on duct size and instrument spacing, in the following table the effects on airflow velocity measurement of an unintentional deviation of up to ±10 mm for the transducers’ axial separation distance, *Z*, and the duct diameter, *D*, or duct height, *H*, are shown. The flow meter scenario used for results in Table 1 is a duct with a diameter or height of 100 mm and a transducer axial separation of 200 mm with a mean airflow of 10 m/s.

**Table 1:** Deviation of *Z*, *D* or *H* on airflow velocity measurement.

Deviation in (mm)		−10	−5	0	5	10
*Z*	Airflow results (m/s)	11.050	10.512	10.000	9.512	9.050
	Deviation from 10 m/s as (%)	10.497	5.125	0.000	−4.875	−9.502
*D*, *H*	Airflow results (m/s)	9.987	9.997	10.000	9.997	9.988
	Deviation from 10 m/s as (%)	−0.131	−0.032	0.000	−0.030	−0.119

The transducer pair axial separation width, *Z*, is fixed at manufacture. This is because as a large error could result from the alteration of this separation distance, as shown in Table 1, where a 5% deviation in separation can cause >10% error in flow measurement. A deviation in diameter or height of the duct of 5% only causes a very small error of <0.05%. This has the benefit that the vertical height of the duct could be affected by a number of factors such as duct mounting arrangements, pressure and bending stresses and the airflow measurement would not change significantly. There is also a possibility that this configuration could be used as a transferable portable device on a number of ducts with different diameters. If, in practice, the circular duct deforms to a slightly elliptical shape, this only causes a small change in the cross-sectional area of the duct so not affecting the volumetric flow rate significantly.

**Figure 4:**
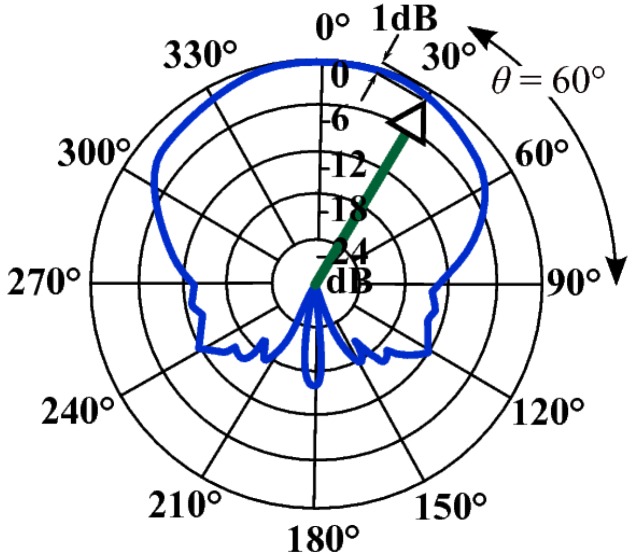
400EP14D enclosed type piezoelectric transducer polar diagram superimposed with the typical acoustic path direction attenuation.

If an ultrasonic transducer with a wide beam width is selected, they can be both mounted level to the printed circuit board so reducing the need for an angled mounting assembly. A reduction in the signal strength is the cost of this mounting arrangement but this is small when using a transducer such as the 400EP14D, which has a −6 dB beam width of 135°, as shown in Figure 4. This only amounts to an extra 2 dB of attenuation for the complete path for both experimental configurations. This also allows a wide range of duct diameters to be accommodated since, as the duct increases, the shortest path direction will move closer to the centre of the transducer beam so reducing the transducer attenuation. The reflective path does not attenuate the signal significantly because 99.99% of the signal energy is reflected because of the high acoustic impedance difference between air and steel [25].

The selection of the horizontal distance, *Z*, needs to be a compromise between sensitivity and the maximum allowable phase difference. The phase difference is the offset in degrees or time between two waveforms having the same frequency. The maximum allowable symmetric phase difference is ±180°, which equates to ±12.5 µs time delay for a transducer frequency of 40 kHz. If a phase difference beyond this range is encountered the velocity reading will wraparound and, for example, a positive reading will then become negative [26]. To avoid this, a margin of around 50% above the maximum typical air velocity was used as the maximum measurable air velocity. Hence the device should typically operate within a phase shift value of ±120° for a symmetrical plus and minus velocity range. The positive airflow velocity range can be increased at the expense of reducing the negative airflow velocity range if negative air velocities are unlikely to be created. The following Equation (10) is used to calculate the typical maximum differential transit time allowable for the ±120° maximum phase shift range specified:

(10) $$\Delta t_{max\;}=\frac{1}{\left(360/120\right)f_{0}}$$

The maximum transducer axial separation distance, *Z_max_*, for the specified maximum differential transit time, Δ*t_max_*, and maximum typical air velocity, *U_upper_*, can be calculated using Equation (11) which is derived from equations described by Lie *et al.* [27]. The transducer separation distance, *Z*, can be shortened to extend the air velocity measurement range but the measurement accuracy could be degraded due to the timing resolution being too large or by the noise on the receive signal.:

(11) $$U_{DTTM}\approx \frac{c^{2}\Delta t}{2Z}\;∴\;Z_{max}\approx \frac{c^{2\;}\Delta t_{max\;}}{2U_{upper}}$$

### 3.3. Ultrasonic Transducer Transmitter Subsystem

**Figure 5:**
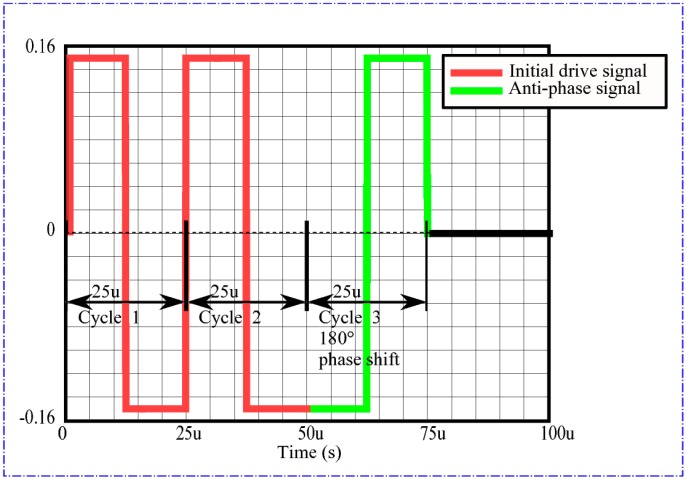
Transducer transmit drive waveform at the output of the AWG.

The transmit signal used was a series of square waves at the transducer operating frequency of 40 kHz, which is less complex to produce and therefore less costly to implement than a sinusoidal waveform. To alleviate problems of receive transducer ringing due to the direct transmission of sound through the duct wall, a self-interference type method [28,29] was used to damp the ringing which is simpler to implement than using active damping [30] or wideband transducers [9,31]. This was done by transmitting a pulse with a 180° phase shift relative to the proceeding pair of pulses. A representation of this transmit drive waveform is shown in Figure 5.

### 3.4. Ultrasonic Transducer Receiver Subsystem

**Figure 6:**
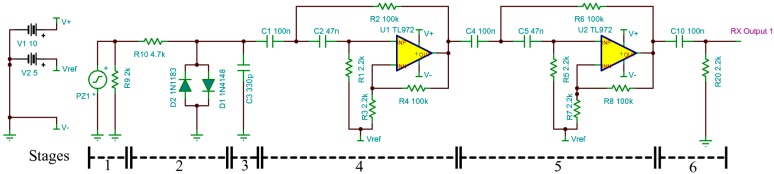
Ultrasonic transducer single channel receiver amplifier circuit diagram.

As shown in Figure 6, the first stage of the receiver circuit consists of a load resistor which the energy from the mainly capacitive ultrasonic transducer is dissipated in, so creating a potential difference. The second stage consists of a current limiting resistor and two signal diodes connected in opposite directions from 0 V to the receiver signal to limit the maximum input voltage to no more than 0.6 V. The current limiting resistor also forms part of the stage 3 resistor capacitor low pass filter with a cut-off frequency of 100 kHz to remove high frequency noise. In the following two stages the signal is amplified by a second-order Sallen-Key high pass filter designed with a stop band set at 100 Hz (−30 dB) and pass band set at 800 Hz. This will give a flat phase response through the transducer in the 40 kHz ± 1 kHz bandwidth frequency range. The design voltage gain of each high pass filter is 50. The overall lower cut-off frequency was 2.3 kHz and the upper was 60.8 kHz with an overall designed voltage gain of 1400 at 40 kHz (in practice a value of 1000 was achieved). It was important to have the high pass filter to remove the 50/60 Hz mains pickup.

The receiver amplifiers were connected to a USB digital oscilloscope which was synchronized by the trigger out signal from the AWG to start sampling the incoming receive signal at a rate of 5 MHz. This data was then transferred to the computer to be processed by the LabVIEW software to provide airflow measurements.

### 3.5. Signal Processing Method

**Figure 7:**
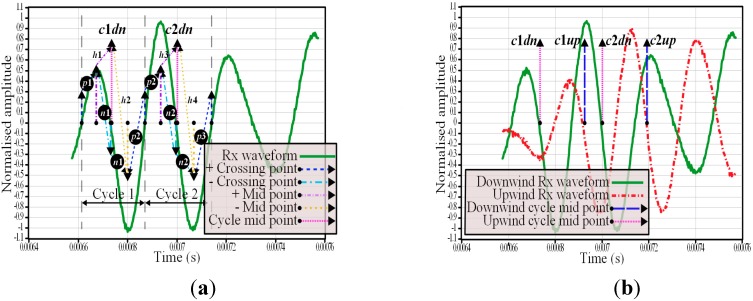
(**a**) Determination of receiver downwind path waveform cycle’s midpoints from zero crossing points; (**b**) Illustration of cycle midpoints for upwind and downwind receiver waveforms.

To determine the time of flight time difference, the zero crossing time and polarity could have been used but there was a problem with the receiver waveform having a fluctuating DC offset which could shift the zero crossing point and cause errors. To alleviate this problem, the time of detection of the positive and negative zero crossing points were recorded from the expected zero flow time of arrival (calculated using Equation (6)) plus a one cycle delay, giving the waveform shown in Figure 7a. At this point the software would start looking for the first positive transition then after this occurrence, would look for the following negative transition and repeat until two cycles had been detected. The next step was to calculate the positive and negative half cycle midpoints which, in turn, would be used to calculate all the whole cycle midpoints (using Equation (12)) as graphically represented in Figure 7a,b:

(12) $$t_{c1dn}=\frac{t_{n1}}{2}+\frac{t_{p1}}{4}+\frac{t_{p2}}{4}$$

The time difference is calculated using Equation (13), by subtracting the first downwind cycle midpoint from the first upwind cycle midpoint, followed by the same for the second cycle midpoints from which the mean of these two results can be calculated:

(13) $$\Delta t_{TR}=\frac{\left(t_{c1dn}-t_{c1up}\right)+\left(t_{c2dn}-t_{c2up}\right)}{2}$$

The mean airflow speed across the centre of the duct can then be calculated by using Equations (5)–(7).

## 4. Experimental Setup

**Figure 8:**
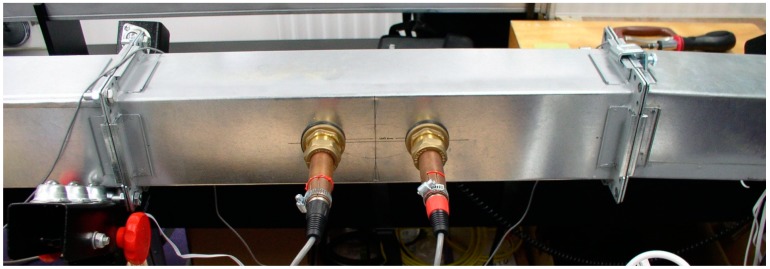
Photograph of 100 mm wide square duct acoustic flowmeter.

The aim of the experiment was to test the ultrasonic duct airflow measurement device as shown in Figure 8 against a Venturi flowmeter over a range of airflow speeds and temperatures.

**Figure 9:**
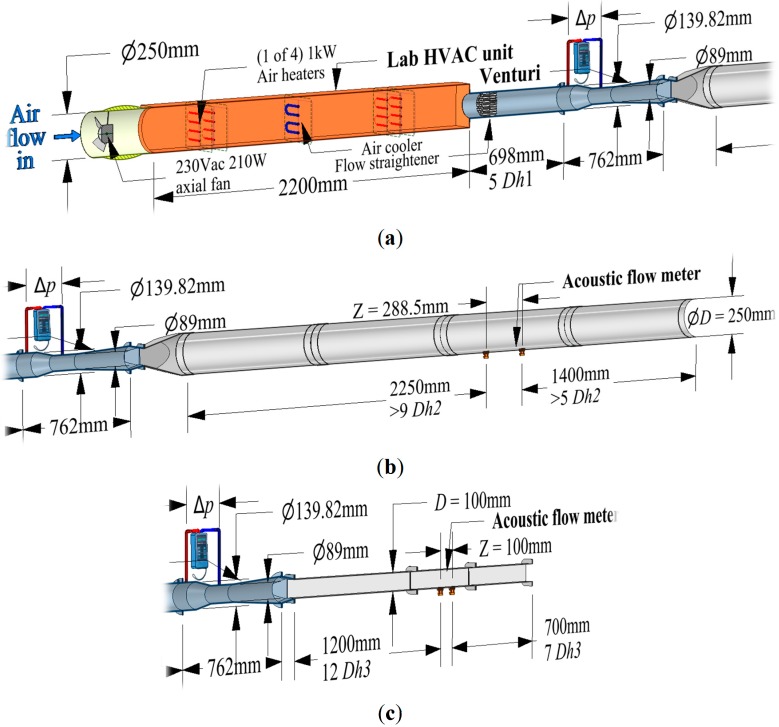
(**a**) Front section of the ultrasonic flowmeter testing rig; (**b**) End section of the cylindrical duct flowmeter testing rig; (**c**) End section of the square duct flowmeter testing rig.

An existing air conditioning laboratory unit with a Venturi meter as shown in Figure 9a was used to provide an adjustable duct airflow rate and temperature in the range 16 to 44 °C for comparison with the acoustic flowmeter device under test. The air conditioning laboratory unit consisted of a 230 V AC 210 W inlet fan to supply air to a duct containing four separate 1 kW heaters and a single cooling heat exchanger. 

The differential pressure across the Venturi was measured by a PVM100 micromanometer (Airflow Developments, High Wycombe, UK). This was connected to the computer running LabVIEW via a serial to USB adapter for calculation of airflow mass and volume rate with corrections for air density variation. To compensate for the assumed Venturi inside surface roughness a discharge coefficient of 0.9877 was used. The test duct lengths before and after the ultrasonic acoustic airflow meter were constructed following recommendations described in the American Society of Heating, Refrigerating and Air Conditioning Engineers (ASHRAE) fundamentals [5] (p. 36.18) for measuring flow in ducts. This requires measurements to be done at least the equivalent of 7.5 hydraulic diameters in length after, and 2 hydraulic diameters before, a disturbance. The hydraulic diameter, *Dh*, of a duct is equivalent to four times the internal cross-sectional area divided by the perimeter length of the duct internal walls [5] (p. 21.7). So for circular or square ducts, the hydraulic diameter is equivalent to the diameter or width of the duct. The Venturi has only five hydraulic diameters before a disturbance but two 50 mm honeycomb flow straighteners are used to mitigate the need for the extra duct length required.

Tests were carried out on a 250 mm diameter cylindrical duct (Figure 9b) then on a 100 mm wide square duct (Figure 8 and Figure 9c) to cover the majority of duct installation types and flow velocities. The negative airflow velocity range was adjusted to a minimum to increase the positive velocity range so that the device would operate at maximum sensitivity for positive flow velocities.

In the first test, a series of airflow rates was produced by varying the inlet fan voltage in steps of 10 V between 120 to 210 V AC. At each voltage step, the Venturi flow rate was recorded against the acoustic flowmeter airflow rate. This test was to check the linearity and the maximum percentage RMS error of the ultrasonic flowmeter compared to the Venturi with varying flow rates. Table 2 lists the operating values of the reference Venturi airflow measurement system for the comparison period time of approximately 1 min.

Table 2(**a**) Operating values for airflow reference system with 250 mm diameter circular duct attached and (**b**) with 100 mm wide square duct attached.

**Table:** 

Inlet Fan (V)	Mean Venturi Air Temp (°C)	Mean Venturi Δ Pressure Readings (Pa)	Standard Deviation of Venturi Pressure Readings (1 sigma)	Calculated Venturi Mean Mass Flow Rate (kg/m^3^)	Calculated Venturi Inlet Flow Velocity (m/s)	Calculated Duct Mean Airflow Velocity (m/s)
**120**	20.9	128	0.35	0.1170	6.44	2.04
**130**	20.9	156	0.63	0.1294	7.12	2.26
**140**	20.8	182	0.50	0.1397	7.68	2.44
**150**	20.7	209	0.42	0.1496	8.23	2.61
**160**	20.6	231	0.43	0.1572	8.65	2.74
**170**	20.6	250	0.78	0.1634	8.99	2.85
**180**	20.6	262	0.68	0.1675	9.22	2.92
**190**	20.5	275	0.40	0.1716	9.44	2.99
**200**	20.3	286	0.72	0.1748	9.61	3.04
**210**	20.2	294	1.04	0.1773	9.75	3.09
(**a**)

**Table:** 

Inlet Fan (V)	Mean Venturi Air Temp (°C)	Mean Venturi Δ Pressure Readings (Pa)	Standard Deviation of Venturi Pressure Readings (1 sigma)	Calculated Venturi Mean Mass Flow Rate (kg/m3)	Calculated Venturi Inlet Flow Velocity (m/s)	Calculated Duct Mean Airflow Velocity (m/s)
**120**	23.1	83	0.43	0.0941	5.21	7.99
**130**	23.2	102	0.29	0.1042	5.77	8.85
**140**	23.2	122	0.45	0.1140	6.31	9.68
**150**	23.2	141	0.45	0.1223	6.77	10.39
**160**	23.1	154	0.00	0.1280	7.08	10.87
**170**	23.2	166	0.21	0.1328	7.35	11.28
**180**	23.2	175	0.21	0.1364	7.55	11.58
**190**	23.2	183	0.00	0.1395	7.72	11.85
**200**	23.3	189	0.29	0.1417	7.84	12.04
**210**	23.2	195	0.49	0.1439	7.96	12.22
(**b**)						

The second test consisted of setting the fan voltage to the maximum step voltage of 210 V AC and measuring the flow rates with different levels of cooling and heating on the air conditioning laboratory unit. This test was to check the maximum percentage RMS error of the ultrasonic flowmeter with a selection of air temperatures between 16 and 44 °C because, as shown in Equation (1), the speed of sound changes with temperature which may lead to errors if temperature corrections for the speed of sound were functioning incorrectly.

## 5. Results and Discussion

**Figure 10:**
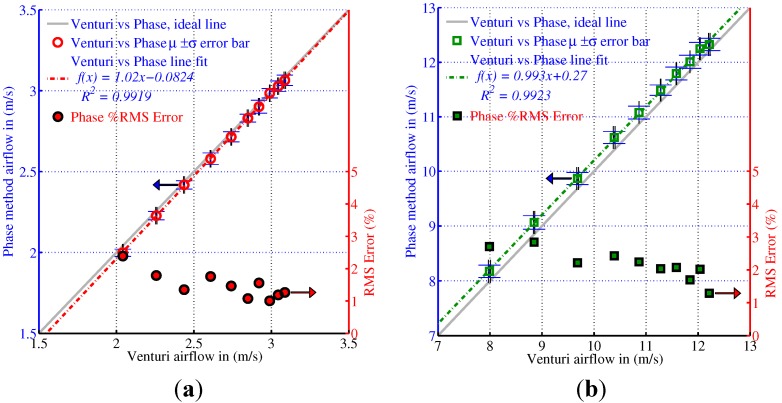

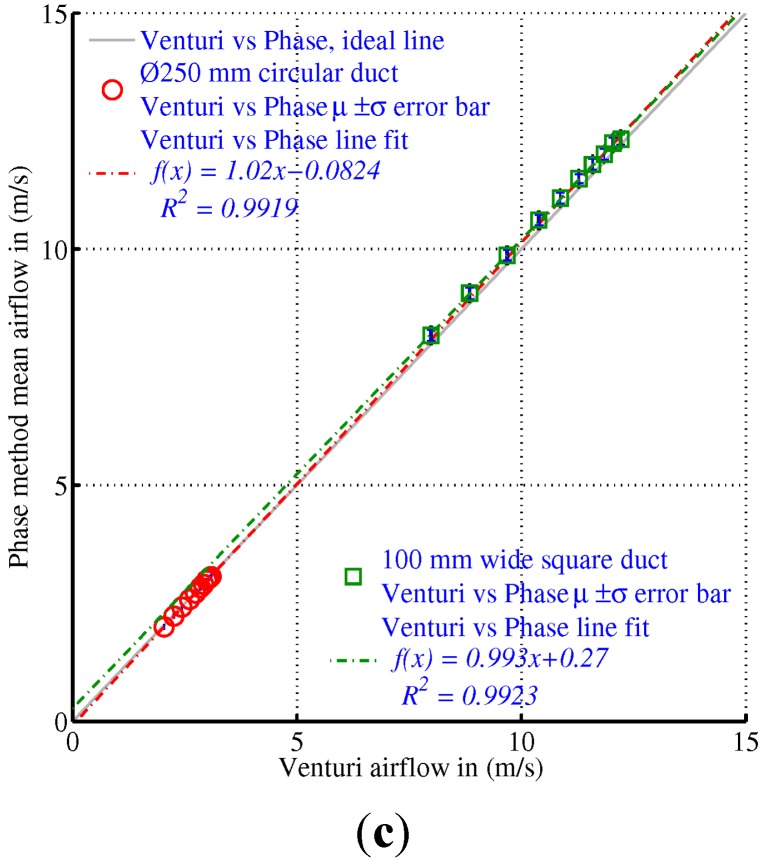
(**a**) 250 mm diameter circular duct ultrasonic airflow measurement *vs.* Venturi; (**b**) 100 mm wide square duct ultrasonic airflow measurement *vs.* Venturi; (**c**) Combined results of circular and square duct airflow measurements *vs.* Venturi.

Figure 10 shows the reference airflow velocity measured by the Venturi against the ultrasonic airflow measurement device under test in a circular and square duct. In the 250 mm diameter circular duct, the range of airflow velocities produced was limited by the output of the inlet fan to between 2 m/s and 3.25 m/s so, in the second set of tests, the smaller size of the duct resulted in a higher range of flow velocities of between 8 m/s and 12.25 m/s. 

**Figure 11:**
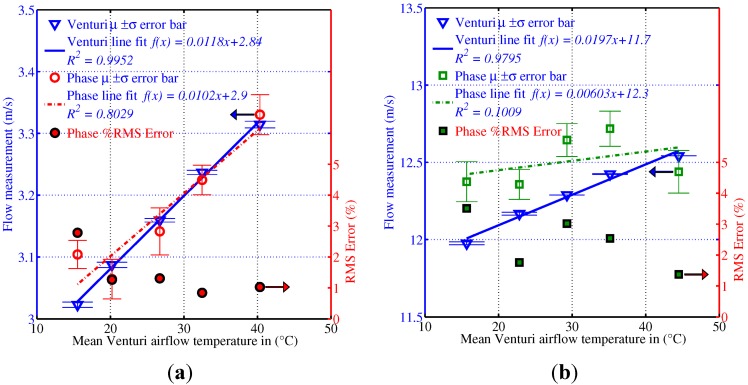
(**a**) 250 mm diameter circular duct ultrasonic airflow measurement *vs.* Venturi with varying air temperature; (**b**) 100 mm wide square duct ultrasonic airflow measurement *vs.* Venturi with varying air temperature.

In both configurations the RMS percentage error was less than 3% with a linear response as shown by the high *R*^2^ values for the straight line fit across the range of air velocities tested. These findings are similar to the results obtained by Olmos [10] and van Buggenhout *et al.* [11]. Figure 11 shows the Venturi velocity against the ultrasonic airflow measurement device under test in a circular and square duct with a range of air temperatures. 

The maximum fan output rate was set on the air conditioning unit and not altered during the tests. There was a small increase in the measured flow rates as the temperature increased due to the expansion of the air in the duct after being heated. In both the circular and square duct scenarios the RMS percentage error was less than 3.5% with no compensation implemented for the error caused by the flow profile shape [32,33]. The low value of *R*^2^ for the results shown in Figure 11b could be due to the overlap of transmitter ringing with the received signal or the signal processing algorithm not having enough robustness to cope with this situation in a small duct. One possible solution to alleviate this would be a “W” shaped acoustic path to double the time of flight.

The design uses a number of techniques and design choices to lower the implementation cost of the device while still providing sufficient measurement quality. The design choices that have been found to work well are the single sided level mounted transducer arrangement for “V” shaped reflective path. The selection of 40 kHz enclosed type transducers was made because of their low cost. The cost of these transducers has fallen, due to mass manufacture to meet everyday use in applications such as car parking proximity sensors. They use a burst of square transmitter pulses which are much easier to implement electrically than the equivalent sine wave waveform. A 180° phase change of the burst pulse train has been used to suppress transducer ringing. The use of the differential transit time method for calculating the approximate time of flight with speed of sound temperature corrections has a much lower cost of implementation. This is because this technique does not have to rely on high-speed analogue to digital converters and intensive digital signal processing but could be implemented using voltage comparators and low-cost microcontrollers.

In the future the design could be further improved by the following:

- Extensive testing on a wider variety of duct sizes and duct shapes with a greater range of airflow velocities.
- Computer modelling of the ultrasonic flowmeter to evaluate its accuracy when it is fitted close to upwind airflow disturbances such as a bend causing duct airflow profile distortion. Test possible solutions to improve on this accuracy, to reduce the amount of expensive laboratory testing needed.
- Experimental testing of the device when fitted in close proximity to disturbances such as bends in different types of duct shape and with turning vanes, where appropriate, and corroborate the computer modelling.
- Extending the function of the device to measure temperature and humidity so that energy efficiency of output can be measured.
- Extending the measurement range of the phase measurement beyond ±180° by using a waveform coarse correlation technique.
- Add corrections required for inaccuracies caused by duct airflow profile.
- Add corrections for humidity change which slightly affect the speed of sound.

## 6. Conclusions/Outlook

In this study, an in-duct ultrasonic airflow measurement device has been designed and tested. The airflow measurement results show that the accuracy is better than 3.5% RMS when used in a 250 mm diameter circular duct and in a 100 mm wide square duct, compared with a Venturi meter over a small range of airflow velocities. The range of the airflow velocities tested (which was limited by the output capacity of the air conditioning laboratory unit used) was 2−3.25 m/s for the 250 mm diameter circular duct and 8–12.25 m/s for the 100 mm wide square duct. The ultrasonic airflow measurement device also had a measurement stability of better than 3.5% RMS for a range of air temperatures between 16 °C and 40 °C.

## Supplementary Materials

Supplementary File 1 includes a document with further design information which is an extended version of the design section included in this article. It can be accessed at: http://www.mdpi.com/1424-8220/15/5/10705/s1.

## List of Symbols

***Symbol***	**Description**	**SI Units**
*c*	Speed of sound	m/s
*D*	Diameter of duct	m
*Dh*	Hydraulic diameters	m
*f_0_*	Transducer centre frequency	Hz
*H*	Height of rectangular duct	m
*L*	Acoustic path length	m
*t*	Air temperature in degrees	°
*t_U0_*	Transit time of acoustic signal in zero flow state	s
*U_m_*	Mean airflow inlet velocity	m/s
*U_DTTM_*	Airflow velocity using phase shift or differential transit time method	m/s
*U_TTM_*	Airflow velocity using transit time method	m/s
*U_upper_*	Typical maximum design air velocity speed	m/s
*Z*	Transducer separation distance with reference to duct axis	m
*Z_max_*	Maximum transducer axial separation distance for airflow measurement range	m
Δ*t_max_*	Typical maximum time of flight difference	s
Δ*t_TR_*	Differential transit time of the contra-propagating acoustic signals	s
*t_TRij_*	Transit time of the acoustic signals between transducers	s
θ	Angle in degrees between acoustic path and the duct wall	°
*t_n1_*	First negative going zero crossing point time, ∴ 2nd is t_n2_	s
*t_p1_*	First positive going zero crossing point time, ∴ 2nd is t_p2_	s
*t_c1dn_*	First downwind cycle midpoint time, ∴ 2nd is t_c2dn_	s
*t_c1up_*	First upwind cycle midpoint time, ∴ 2nd is t_c2up_	s

## References

[1] Yu D., Li H., Yang M. (2011). A virtual supply airflow rate meter for rooftop air-conditioning units. Build. Environ..

[2] Cui J., Wang S. (2005). A model-based online fault detection and diagnosis strategy for centrifugal chiller systems. Int. J. Therm. Sci..

[3] Dexter A., Pakanen J. Fault detection and diagnosis methods in real buildings. http://www.ecbcs.org/annexes/annex34.htm.

[4] Yu Y., Woradechjumroen D., Yu D. (2014). A review of fault detection and diagnosis methodologies on air-handling units. Energy Build..

[5] (2009). ASHRAE Handbook, 2009, Fundamentals.

[6] Lynnworth L.C., Liu Y. (2006). Ultrasonic flowmeters: Half-century progress report, 1955–2005. Ultrasonics.

[7] Swengel R.C. (1956). Fluid Velocity Measuring System. U.S. Patent.

[8] Gas Flow Measuring Devices. http://www.sick.com/uk/en-uk/home/products/product_portfolio/flow_solutions/Pages/flow_devices.aspx.

[9] Tauch M., Witrisal K., Kudlaty K., Noehammer S., Wiesinger M. System identification method for Ultrasonic Intake Air Flow Meter for engine test bed applications. Proceedings of the 2012 IEEE International Instrumentation and Measurement Technology Conference (I2MTC).

[10] Olmos P. (2004). Ultrasonic velocity meter to evaluate the behaviour of a solar chimney. Meas. Sci. Technol..

[11] Van Buggenhout S., Ozcan S.E., Vranken E., van Malcot W., Berckmans D. Acoustical ventilation rate sensor concept for naturally ventilated buildings. ASHRAE Transactions; Proceedings of of the American-Society-of-Heating-Refrigerating-and-Air-Conditioning-Engineers.

[12] Bragg M.I., Lynnworth L.C. Internally-nonprotruding one-port ultrasonic flow sensors for air and some other gases. Proceedings of the International Conference on Control.

[13] Rabalais R.A., Sims L. Ultrasonic Flow Measurement: Technology and Applications in Process and Multiple Vent Stream Situations. https://www.idc-online.com/technical_references/pdfs/instrumentation/Ultrasonic_Flow_Measurement.pdf.

[14] Strauss J., Weinberg H., Kopel Z. (1996). Ultrasound Air Velocity Detector for HVAC Ducts and Method Therefor 1996. U.S. Patent.

[15] Olmos P. (2002). Extending the accuracy of ultrasonic level meters. Meas. Sci. Technol..

[16] Conrad K., Lynnworth L. Fundamentals of Ultrasonic Flow Meters. http://asgmt.com/paper/fundamentals-of-ultrasonic-flow-meters-2002/.

[17] Vermeulen M.J.M., Drenthen J.G., den Hollander H. Understanding Diagnostic and Expert Systems in Ultrasonic Flow Meters. http://asgmt.com/paper/understanding-diagnostic-and-expert-systems-in-ultrasonic-flow-meters-2012/.

[18] Bohn D.A. (1988). Environmental Effects on the Speed of Sound. J. Audio Eng. Soc..

[19] Han D., Kim S., Park S. (2008). Two-dimensional ultrasonic anemometer using the directivity angle of an ultrasonic sensor. Microelectron. J..

[20] Hirata S., Kurosawa M.K., Katagiri T. Real-time ultrasonic distance measurements for autonomous mobile robots using cross correlation by single-bit signal processing. Proceedings of the IEEE International Conference on Robotics and Automation.

[21] Hofmann F. Fundamentals of Ultrasonic-Flow Measurement for Industrial Applications. http://www.investigacion.frc.utn.edu.ar/sensores/Caudal/HB_ULTRASONIC_e_144.pdf.

[22] Chen Q., Li W., Wu J. (2014). Realization of a multipath ultrasonic gas flowmeter based on transit-time technique. Ultrasonics.

[23] De Cicco G., Morten B., Prudenziati M., Taroni A., Canali C. A 250 KHz Piezoelectric Transducer for Operation in Air: Application to Distance and Wind Velocity Measurements. Proceedings of the 1982 Ultrasonics Symposium.

[24] Quaranta A.A., Aprilesi G.C., Cicco G.D., Taroni A. (1985). A microprocessor based, three axes, ultrasonic anemometer. J. Phys. E: Sci. Instrum..

[25] Bentley J.P. (2005). Principles of Measurement Systems.

[26] Han D., Park S. (2011). Measurement range expansion of continuous wave ultrasonic anemometer. Measurement.

[27] Lie I., Tiponut V., Bogdanov I., Ionel S., Caleanu C.D. The Development of CPLD-based Ultrasonic Flowmeter. Proceedings of the 11th WSEAS International Conference on Circuits.

[28] Cai C.H., Regtien P.P.L. (1993). Accurate Digital Time-of-Flight Measurement Using Self-Interference. IEEE Trans. Instrum. Meas..

[29] Ji-De H., Chih-Kung L., Chau-Shioung Y., Wen-Jong W., Chih-Ting L. (2011). High-Precision Ultrasonic Ranging System Platform Based on Peak-Detected Self-Interference Technique. IEEE Trans. Instrum. Meas..

[30] Miller G.L., Boie R.A., Sibilia M. Active damping of ultrasonic transducers for robotic applications. Proceedings of the 1984 IEEE International Conference on Robotics and Automation.

[31] Buess C., Pietsch P., Guggenbühl W., Koller E.A. (1986). Design and Construction of a Pulsed Ultrasonic Air Flowmeter. IEEE Trans. Biomed. Eng..

[32] Baker R.C. (2000). Flow Measurement Handbook: Industrial Designs, Operating Principles, Performance, and Applications.

[33] Jung J.C., Seong P.H. (2005). Estimation of the flow profile correction factor of a transit-time ultrasonic flow meter for the feedwater flow measurement in a nuclear power plant. IEEE Trans. Nucl. Sci..

