# Supplementary material for: Development of an Ultrasonic Airflow Measurement Device for Ducted Air
Source: Sensors (Basel). 2015 May 6;15(5):10705–22. doi: 10.3390/s150510705 (PMC4481905; doi:10.3390/s150510705)
Supplement: Supplementary file 1 [file sensors-15-10705-s001.pdf]

OPEN ACCESS
sensors
ISSN 1424-8220
www.mdpi.com/journal/sensors

Supplementary Information

Development of an Ultrasonic Airflow Measurement Device for Ducted Air. *Sensors* 2015, *15*, 10705-10722

Andrew B. Raine, Nauman Aslam *, Christopher P. Underwood and Sean Danaher

Faculty of Engineering and Environment, Northumbria University, Newcastle upon Tyne NE18ST, UK; E-Mails: andrew.raine@northumbria.ac.uk (A.B.R.); chris.underwood@northumbria.ac.uk (C.P.U.); sean.danaher@northumbria.ac.uk (S.D.)

* Author to whom correspondence should be addressed; E-Mail: nauman.aslam@northumbria.ac.uk; Tel.: +44-191-243-7737; Fax: +44-191-243-7630.

1. Ultrasonic Airflow Measurement Device for Ducted Air Further Supplementary Design Information Document

1.1. HVAC Ultrasonic Duct Airflow Measurement Development System Overview

An ultrasonic duct airflow development system as illustrated in Figure S1 below was produced which had the flexibility to test various measurement techniques.

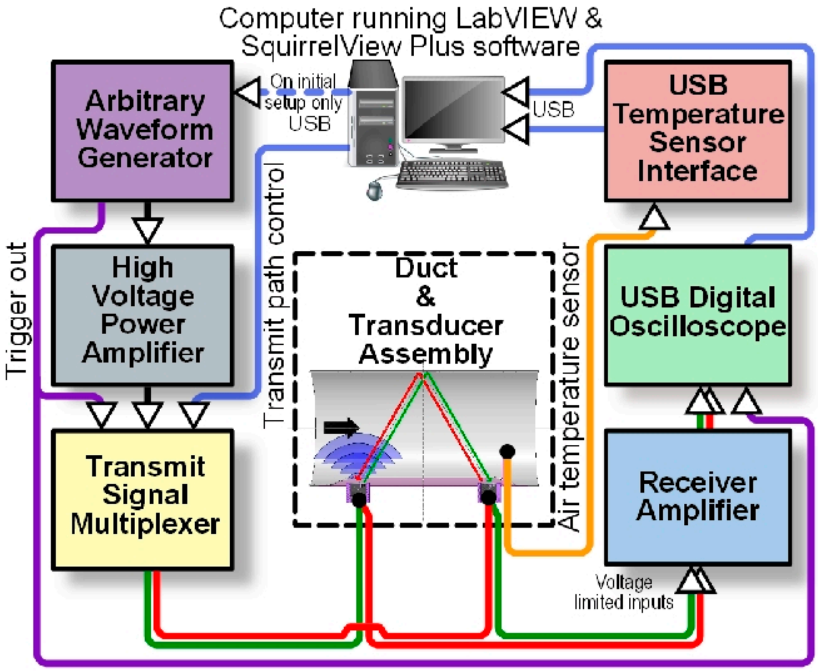

Figure S1. Schematic of the ultrasonic duct airflow measuring device development system.

A Tektronix AFG3021C 25 MHz single channel computer controlled arbitrary waveform generator (AWG) was used so that any type of transmit drive signal could be produced and manipulated quickly by the extensively modified LabVIEW® 10 (National Instruments, Austin, TX, USA) instrument driver software. The output of the AWG was fed into a PA95 (Apex Microtechnology, Tucson, AZ, USA) high voltage power amplifier capable of driving the ultrasonic transducer to its maximum voltage of approximately 100 Volts peak to peak. This was then switched through two LH1500AT (Vishay Intertechnology, Malvern, PA, USA) solid-state relays (SSR) within the high voltage multiplexer specifically designed for the task which could switch a single input between at least two outputs. The multiplexer was also controlled by the computer through a PicoLog® 1012 (Pico Technology, St Neots, UK) universal serial bus (USB) data acquisition device. The transmit signals were then connected, depending on the multiplexer state, to one of two 400EP14D (Pro-Wave Electronic Corp., New Taipei City, Taiwan) 40 kHz enclosed type piezoelectric transducers which would transmit an ultrasonic signal through the duct to a receiving transducer. The received signal would be then amplified after passing through a diode voltage limiter input circuit to a multistage operational amplifier to boost the signal voltage gain to >1000. This was then digitized by a USB oscilloscope, which is shown in Figure S2, for processing by the computer running the LabVIEW® control and signal processing software.

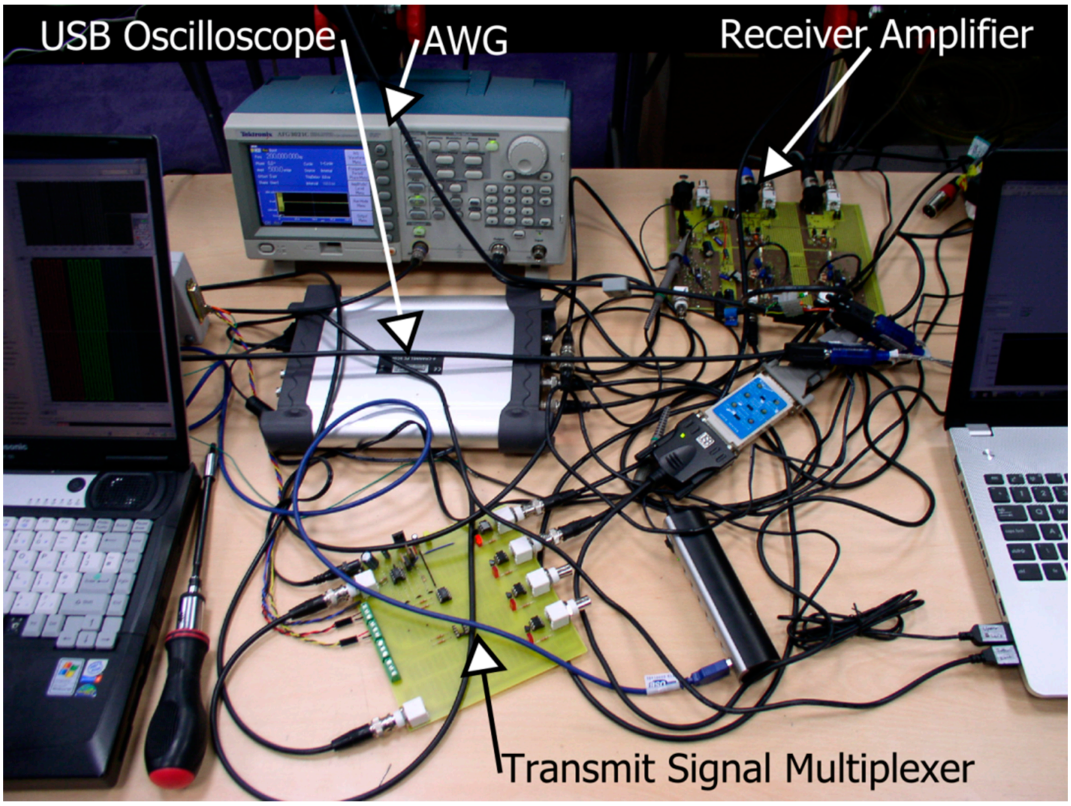

Figure S2. Photograph of development system hardware excluding the temperature sensor interface, high voltage power amplifier and transducer assembly.

A Grant type U thermistor probe was used to measured the in duct air temperature and was monitored via a Squirrel® SQ2020 Series Data Logger (Grant Instruments, Shepreth, UK).

1.2. HVAC Ultrasonic Duct Airflow Measurement Instrument Description

1.2.1. Transducer Configuration

A single reflective path ultrasonic flow meter design was chosen as the preferred solution. The transducers are mounted on the same side so that they can be constructed as a single assembly which is fitted to the duct wall. This should reduce the overall cost as it simplifies installation and reduces the number of cable assemblies required.

The single reflective "V" shaped path geometric parameter values is given by the following Equations (1) and (2):

$$L = \sqrt{2D^2 + Z^2} \quad (1)$$

$$\theta = \tan^{-1}\frac{2D}{Z} \quad (2)$$

Figure S3 shows a representation of an ultrasonic flow meter design with a reflective "V" shaped path with the transducers mounted perpendicular to the duct wall.

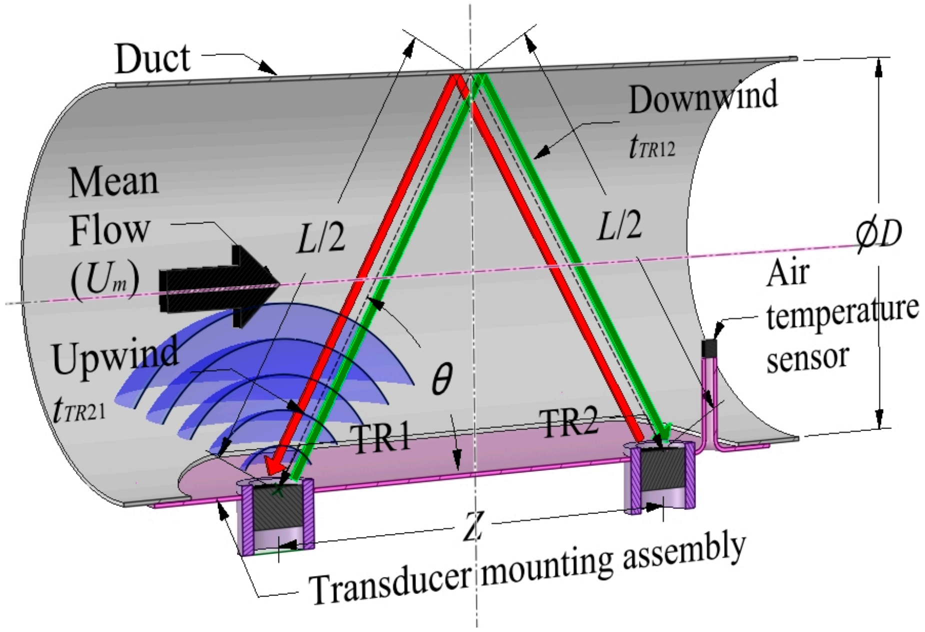

Figure S3. A reflective "V" shape single path acoustic differential transit time flow meter in a cylindrical duct.

To explore the sensitivity of airflow measurements on duct size and instrument spacing, in the following table the effects on airflow velocity measurement of unintentional deviation of up to ±10 mm for the transducers axial separation distance *Z* and the duct diameter *D* or duct height *H* are shown. The flow meter scenario used for results in Table S1 is a duct with a diameter or height of 100 mm and a transducer axial separation of 200 mm with a mean airflow of 10 m/s.

Table S1. Deviation of *Z* or *D*,*H* on airflow velocity measurement.

	Deviation in (mm)	−10	−5	0	5	10
Z	Airflow results (m/s)	11.050	10.512	10.000	9.512	9.050
	Deviation from 10 m/s as (%)	10.497	5.125	0.000	−4.875	−9.502
D,*H*	Airflow results (m/s)	9.987	9.997	10.000	9.997	9.988
	Deviation from 10 m/s as (%)	−0.131	−0.032	0.000	−0.030	−0.119

The transducer pair axial separation width *Z* is fixed at manufacture as a large error could be cause by the alteration of this separation distance as shown in Table S1 where a 5% deviation in separation can cause >10% error in flow measurement. A deviation in diameter or height of the duct of 5% only causes a very small error of <0.05%. This has the benefit that the vertical height of the duct could be affected by a number of factors such as duct mounting arrangements, pressure and bending stresses and the airflow measurement would not change significantly. There is also a possibility that this configuration could be used as a transferable portable device on a number of ducts with different diameters. If, in practice, the circular duct deforms to a slightly elliptical shape, this only causes a small change in the cross-sectional area of the duct so not affecting volumetric flow rate significantly.

If an ultrasonic transducer with a wide beam width is selected they can be both mounted level to the printed circuit board so reducing the need for an angled mounting assembly. A reduction in the signal strength is the cost of this mounting arrangement but this is small when using a transducer such as the 400EP14D, which has a −6 dB beam width of 135°, as shown in Figure S4 which only amounts to an extra 2 dB of attenuation for the complete path for both the experimental configurations. This also allows a wide range of duct diameters to be accommodated as the duct increases the shortest path direction will move closer to the centre of the transducer beam so reducing the transducer attenuation. The reflective path does not attenuate the signal significantly because 99.99% of the signal energy is reflected because of the high acoustic impedance difference between air and steel [1].

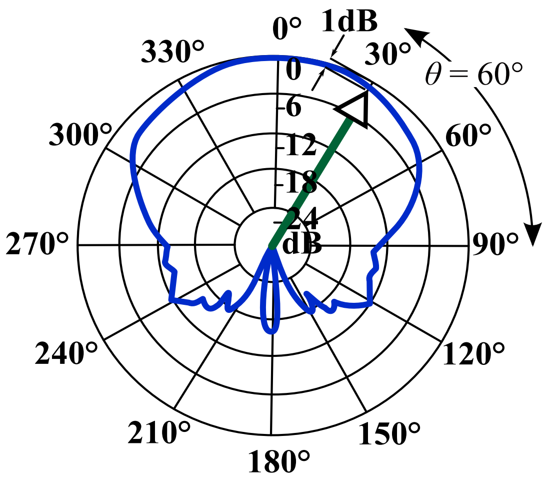

Figure S4. 400EP14D enclosed type piezoelectric transducer polar diagram superimposed with the typical acoustic path direction attenuation.

To reduce errors caused by noise and fluctuating signal levels the absolute transit time is usually measured by using the cross-correlation [2] digital signal processing (DSP) method [3] which

compares the digital representation of the transmit and received waveforms to calculate the delay between them.

In this study a phase shift or differential transit time method similar to that described by Han *et al.* [4] and de Cicco *et al.* [5] is used to reduce the cost of implementation for this application. In the differential transit time method the estimated transit time is found by using a two stage calculation. In the first stage, the zero flow transit time is calculated with Equation (3). In this equation the acoustic path length in Equation (1) is divided by the speed of sound, which is derived from the airflow temperature.

$$t_{U0} = \frac{L}{c} \tag{3}$$

In the second stage, half the actual measured differential transit time is added or subtracted from the zero flow transit time depending on the airflow direction, which is represented in the Equation (4) below.

$$t_{TR} = t_{U0} \pm \frac{\Delta t_{TR}}{2} \tag{4}$$

The airflow velocity can then be calculated by using the inverse transit time difference (ITTD) [6] formula.

The selection of the transducers axial separation distance *Z* needs to be a compromise between sensitivity and the maximum allowable phase difference. The phase difference is the offset in degrees or time between two waveforms having the same frequency. The maximum allowable symmetric phase difference is ±180°, which equates to ± 12.5 μs time delay for a transducer frequency of 40 kHz, if a phase difference beyond this range is encountered the velocity reading will wraparound and for example a positive reading will then become negative. To avoid this, a margin of about 50% above the maximum typical air velocity was used as the maximum measurable air velocity. Hence the device should typically operate within a phase shift value of ±120° for a symmetrical plus and minus velocity range. The positive airflow velocity range can be increased at the expense of reducing the negative airflow velocity range if negative air velocities are unlikely to be created. The following Equation (5) is used to calculate the typical maximum differential transit time allowable for the ±120° maximum phase shift range specified.

$$\Delta t_{max} = \frac{1}{(360/120)f_0} \tag{5}$$

The maximum transducer axial separation distance Z_{max} for the specified maximum differential transit time Δt_{max} and maximum typical air velocity U_{upper} can be calculated using Equation (6) below which is derived from equations described by Lie *et al.* [7]. The transducer separation Z distance can be shortened to extend the air velocity measurement range but the measurement accuracy could be degraded by timing resolution being too large or by noise on the receive signal.

$$U_{DTTM} \approx \frac{c^2 \Delta t}{2Z} \therefore Z_{max} \approx \frac{c^2\, \Delta t_{max}}{2U_{upper}} \tag{6}$$

1.2.2. Ultrasonic Transducer Transmitter Subsystem

The output of the voltage of the AWG was amplified 100 times by the APEX PA95 very high power operational amplifier. As shown in Figure S5 below this was then fed through a SSR device with the capacity to handle very high voltages and also have a much higher switching life compared to electromechanical relays.

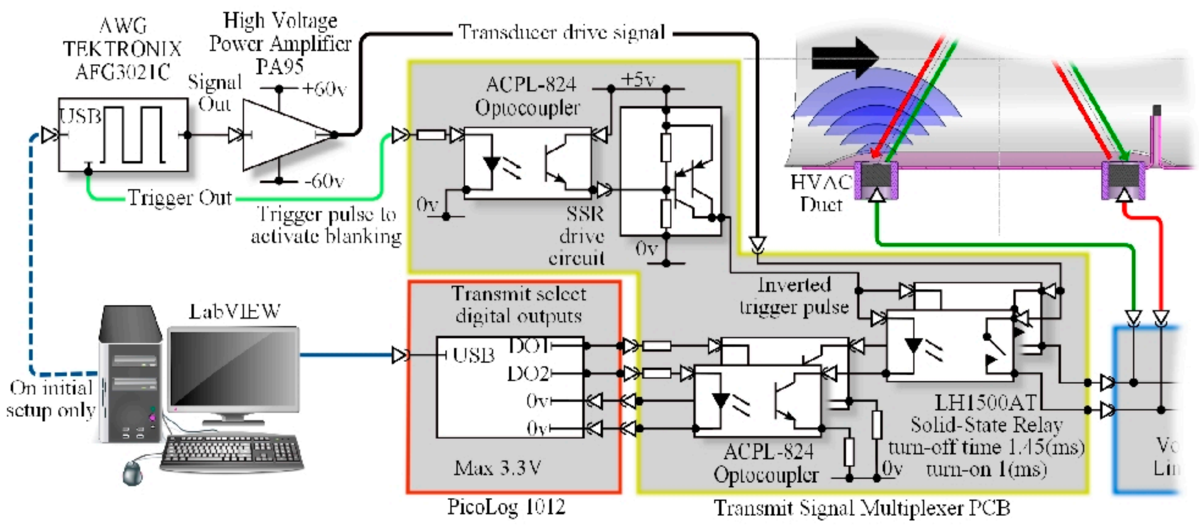

Figure S5. Overview schematic of the ultrasonic transducer transmitter subsystem.

In the selection of the SSR device the output off capacitance value [8] was a important consideration because if it was too high the high frequency noise from the high voltage power amplifier would pass through the device even in the off state which would increase the noise level into the receiver amplifiers. The transmit path was controlled by LabVIEW and a PicoLog 1012 which would enable the SSR device when required. The SSR device chosen had a turnoff delay of about 1.5 ms which was used as the switch off delay from the falling edge of the inverted AWG trigger out. Hence, as soon as the transmitter pulses were generated the SSR would start switching off but by the time it had actually turned off the pulses had passed through the device and the system was now ready for reception of the reflected ultrasonic pulses.

The transmit signal used was a series of square waves at the transducer operating frequency of 40 kHz. To alleviate problems with the receive transducer ringing because of the direct transmission of sound through the duct wall a self interference type method [9,10] was used to damp the ringing. This was done by transmitting a pulse with a 180° phase shift relative to a pair transmitted previously with no delay between them, as shown in Figure S6 below.

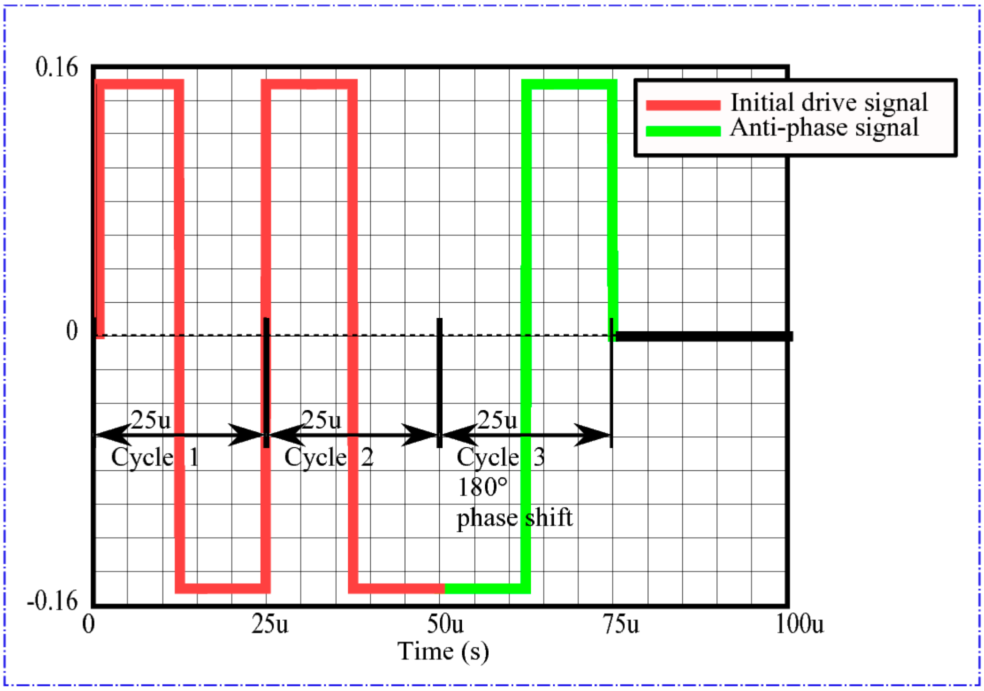

Figure S6. Transducer transmit drive waveform.

This reduced the ringing to some extent and enable a higher measurement rate to be used but was still not enough for the 100 mm wide square duct because of the short time of flight. It was found that by pressing likely on the top surface of the transducers with the point of a finger the ringing could be suppressed. It was then found that the finger could be replaced with a piece of reusable mounting putty similar to Blu Tack®, which was moulded in a dish like shape as shown in Figure S7.

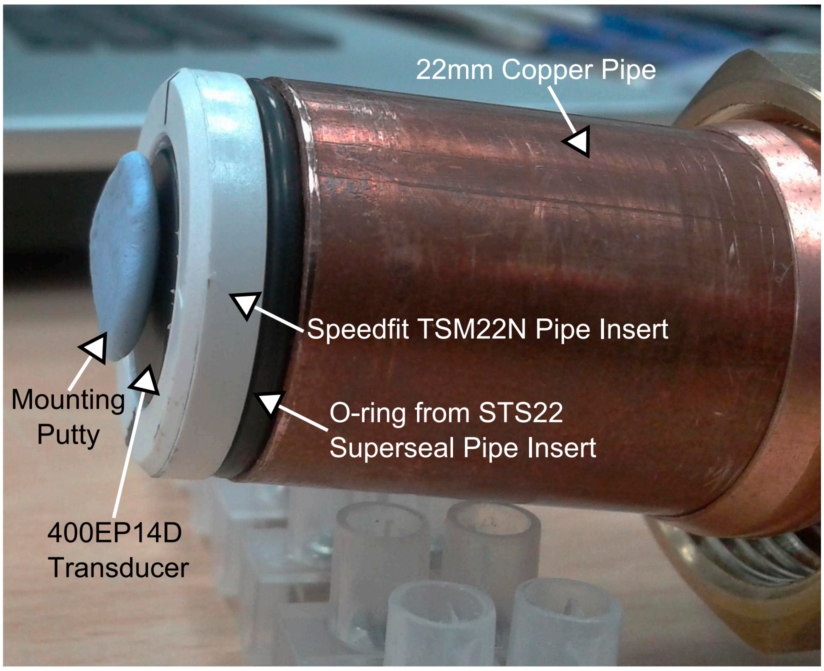

Figure S7. Photograph of transducer mounting arrangement and putty used to damp ringing in 100 mm square duct tests.

This seemed to work best when the contact point was as small as possible without the putty being able to fall off. As this was not a solution that could be used permanently a “W” acoustic path was used later after the results in the study for the square duct had been taken. The signal would reflect twice off the adjacent duct wall giving more time for the ringing to dwindle. When the “W” acoustic path was used the parameter *D* was doubled to take this into account. An active transducer damping circuit as described by Miller *et al.* [11] was also a possible solution for this problem but has not been tested in this study.

The transducer was mounted as shown in Figure S8, into a John Guest® 22 mm TSM22N Speedfit® pipe insert using the same putty as mentioned before to fill the gap between the transducer and the inside of the pipe insert.

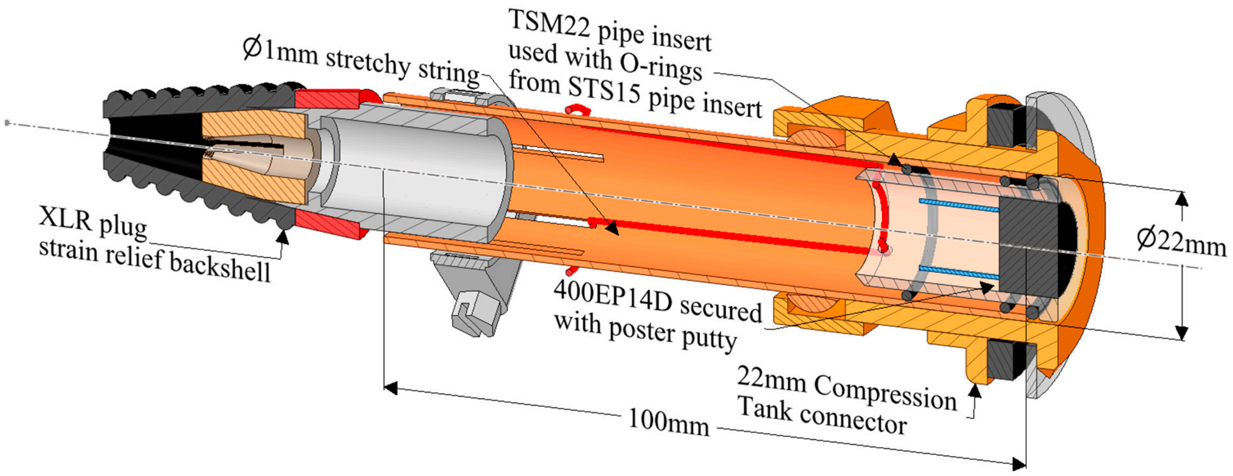

Figure S8. Ultrasonic transducer mounting system for acoustic/vibration isolation.

Three rubber O-rings were used to provide vibration isolation between the pipe insert and the 22 mm copper pipe. The O-rings were taken from John Guest® 22 mm STS22 Speedfit® superseal pipe inserts which each have two O-rings which one is slightly smaller than the other. At least two of the smaller O-rings were used per transducer mounting. The pipe insert was forced against one of the larger O-ring by the use of elastic beading wire connected to the other end of the copper pipe. The back shell of a XLR type electrical connector, which was secured by a hose clamp was used to provide cable strain relief to protect the delicate connections of the 400EP14D ultrasonic transducer. The transducer assembly was then attached to the duct with a 22 mm compression flanged tank connector. The tank connector had to have a few modifications which one was to allow the 22 mm pipe to travel all the way through the connector, which was done on a lathe.

The other modification was to chamfer the edges of the connector flange as shown in Figure S9, so that it would create less turbulence and airflow resistance inside the duct. Two opposite sides of the chamfered flange was removed using a milling machine so that it would fit flush in a circular duct and not distort the duct when the tank connector nut was tightened.

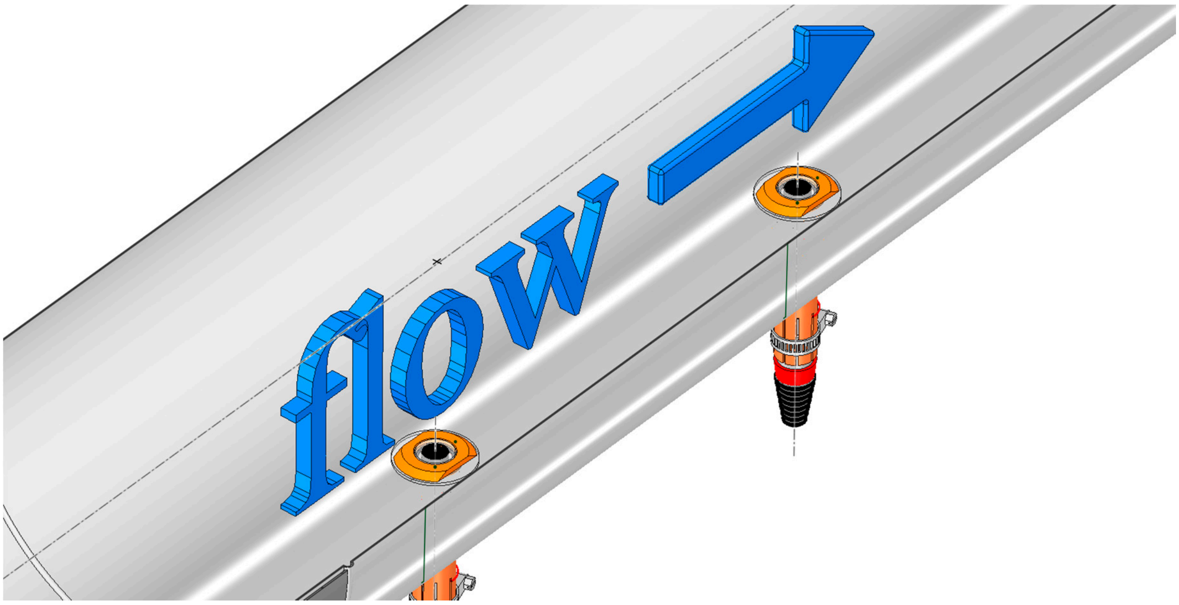

Figure S9. Pair of Ultrasonic transducer assemblies fitted to a circular duct.

1.2.3. Ultrasonic Transducer Receiver Subsystem

As shown in Figure S10 the first stage of the receiver circuit consists of a load resistor which the energy from the mainly capacitive ultrasonic transducer is dissipated in, so creating a potential difference. The second stage consists of a current limiting resistor and two signal diodes connected in opposite directions from 0 V to the receiver signal to limit the maximum input voltage to no more than 0.6 V. The current limiting resistor also forms part of the stage 3 resistor capacitor low pass filter with a cut-off frequency of 100 kHz to remove high frequency noise. In the following two stages the signal is amplified by a second-order Sallen-Key high pass filter designed with a stop band set at 100 Hz (−30 dB) and pass band set at 800 Hz to give a flat phase response through the transducer in the 40 kHz ± 1 kHz bandwidth frequency range. The design voltage gain of each high pass filter is 50. The overall lower cut-off frequency was 2.3 kHz and the upper was 60.8 kHz with an overall designed voltage gain of 1400 at 40 kHz (in practice a value of 1000 was achieved). It was important to have the high pass filter to remove the 50/60 Hz mains pickup. The output of the final operational amplifier stage was then connected in series to a capacitor to remove the DC component. It was found that if the output of this stage was not loaded by a resistor to 0 V the amplifier would start to oscillate when triggered by an external electrical noise event.

As shown in Figure S11 below the output of the receiver amplifiers were connected to a USB digital oscilloscope which was synchronized by the trigger out signal from the AWG to start sampling the incoming receive signal at a rate of 5 MHz. This data was then transferred to the computer to be processed by the LabVIEW software to provide airflow measurements.

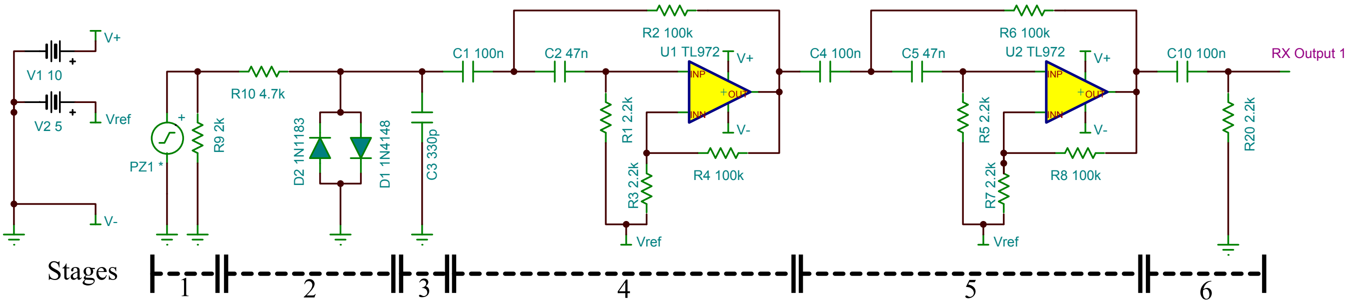

Figure S10. Ultrasonic transducer single channel receiver amplifier circuit diagram.

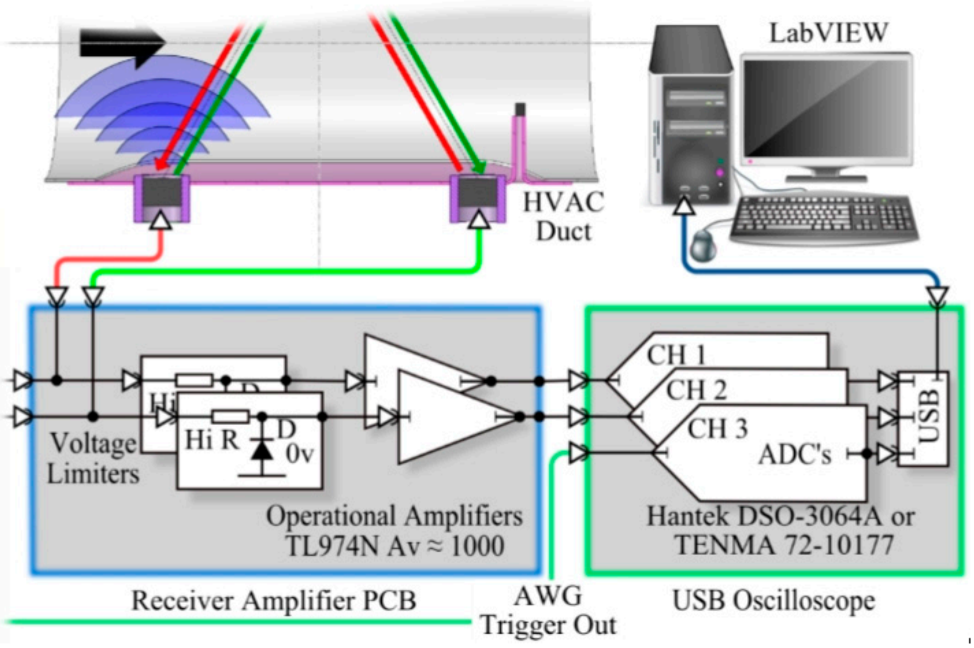

Figure S11. Overview schematic of the ultrasonic transducer receiver subsystem.

1.2.4. Signal Processing Method

To determine the time of flight time difference the zero crossing time and polarity could have been used but there was a problem with the receiver waveform having a fluctuating DC offset which could shift the zero crossing point and cause errors. To alleviate this problem the positive and negative zero crossing points time of detection were recorded from the expected zero flow time of arrival which was calculated by Equation (3) plus a one cycle delay which gave the waveform shown in Figure S12. At this point the software would start looking for the first positive transition then after this occurrence, would look for the following negative transition and repeat until two cycles had been detected. The next step was to calculate the positive and negative half cycle midpoints which in turn would be used to calculate all the whole cycle midpoint as shown in Figure S13 and by using Equation (7).

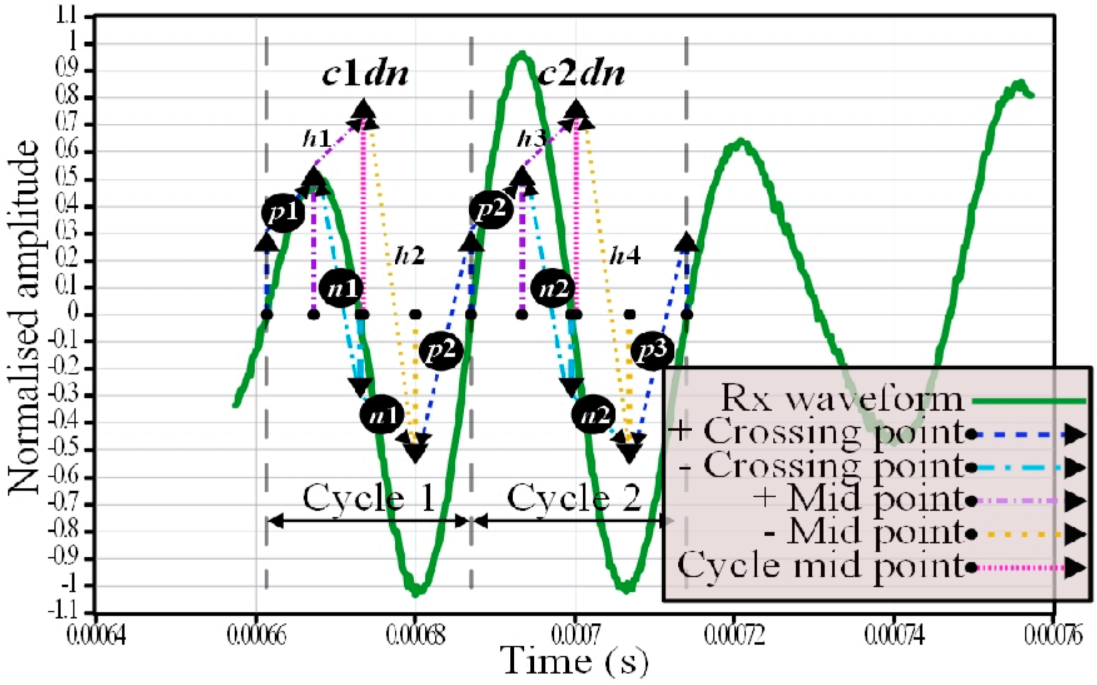

Figure S12. Determination of receiver downwind path waveform cycle's midpoints from zero crossing points.

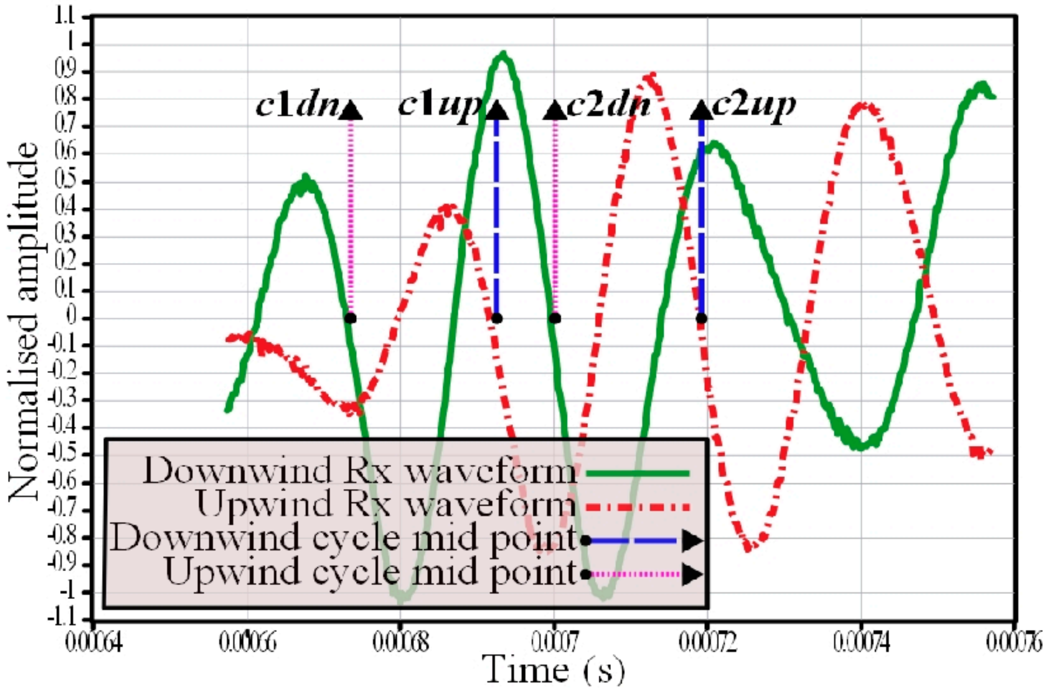

Figure S13. Illustration of cycle midpoints for upwind and downwind receiver waveforms.

$$t_{c1dn} = \frac{t_{n1}}{2} + \frac{t_{p1}}{4} + \frac{t_{p2}}{4} \tag{7}$$

The time difference is calculated using Equation (8), by subtracting the first downwind cycle midpoint from the first upwind cycle midpoint and then the same for the second cycle midpoint from which the mean of these two results can be calculated.

$$\Delta t_{TR} = \frac{(t_{c1dn} - t_{c1up}) + (t_{c2dn} - t_{c2up})}{2} \quad (8)$$

The mean airflow speed across the centre of the duct can then be calculated.

1.2.5. Duct Air Temperature Subsystem

The air temperature was measured with a Grant type U thermistor part number FF-U-VS-0 which was placed at about half the radius into the duct to avoid the slight temperature change that occurs near the duct wall because of the internal and external duct air temperature difference. The thermistor accuracy was reported to be ±0.2 °C for the temperature range 0 °C to 70 °C. The Grant 2020 datalogger which the thermistor was connected to as shown in Figure S14 was configured to send temperature information to a computer running the Grant SquirrelView Plus software. On another computer running LabVIEW the messages from the datalogger containing the temperature information which was formatted in a sequence of 32-bit single-precision, floating-point numbers starting from the eighth byte were able to be read. This was done because a Grant data logger purchased by the University was available to use but National Instruments (NI) hardware required would have incurred extra expense but would of been much easier to interface to. The thermistor was chosen because of its higher accuracy in the ambient air temperature range compared to thermocouple devices.

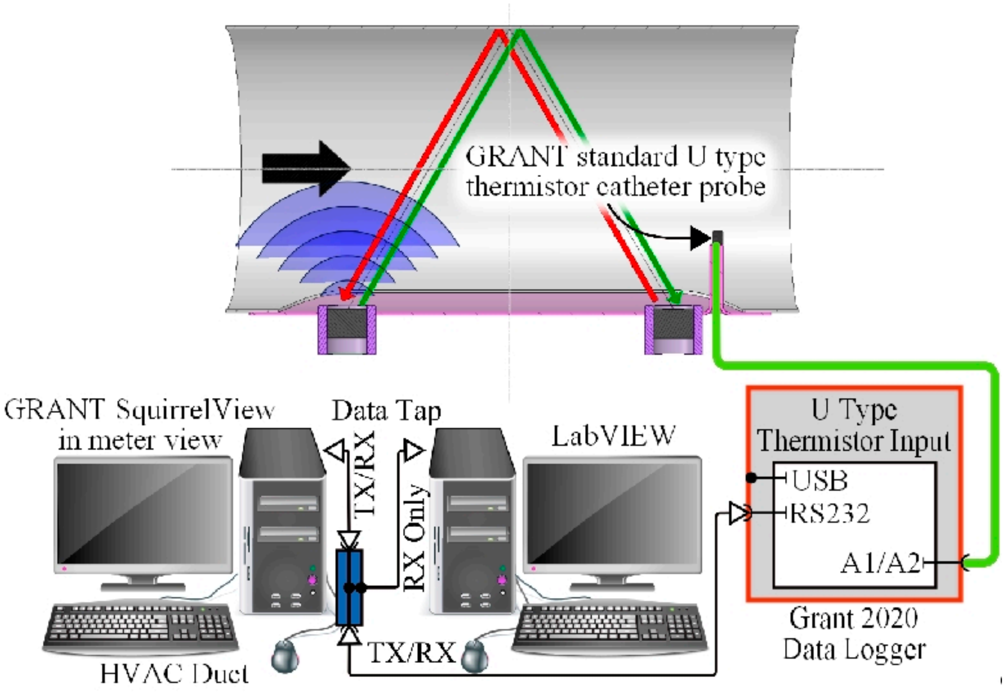

Figure S14. Overview schematic of the air temperature subsystem.

References

1. Bentley, J.P. *Principles of Measurement Systems*, 4th ed.; Pearson/Prentice Hall: Harlow, UK, 2005; pp. 439–443.
2. Hirata, S.; Kurosawa, M.K.; Katagiri, T. Real-time ultrasonic distance measurements for autonomous mobile robots using cross correlation by single-bit signal processing. In Proceedings of the IEEE International Conference on Robotics and Automation, Kobe, Japan, 12–17 May 2009; pp. 3601–3606.
3. Hofmann, F. Fundamentals of Ultrasonic-Flow Measurement for Industrial Applications. Available online: http://www.investigacion.frc.utn.edu.ar/sensores/Caudal/HB_ULTRASONIC_e_144.pdf (accessed on 12 September 2014).
4. Han, D.; Kim, S.; Park, S. Two-dimensional ultrasonic anemometer using the directivity angle of an ultrasonic sensor. *Microelectron. J.* **2008**, *39*, 1195–1199.
5. De Cicco, G.; Morten, B.; Prudenziati, M.; Taroni, A.; Canali, C. A 250 KHz Piezoelectric Transducer for Operation in Air: Application to Distance and Wind Velocity Measurements. In Proceedings of the 1982 Ultrasonics Symposium, San Diego, CA, USA, 27–29 October 1982; pp. 321–324.
6. Quaranta, A.A.; Aprilesi, G.C.; Cicco, G.D.; Taroni, A. A microprocessor based, three axes, ultrasonic anemometer. *J. Phys. E: Sci. Instrum.* **1985**, *18*, 384.
7. Lie, I.; Tiponut, V.; Bogdanov, I.; Ionel, S.; Caleanu, C.D. The Development of CPLD-based Ultrasonic Flowmeter. In Proceedings of the 11th WSEAS International Conference on Circuits, Agios Nikolaos, Crete Island, Greece, 23–25 July 2007; pp. 190–193.
8. AVAGO; Low On-Resistance Solid State Relays; Application Note 1046, Available online: http://www.avagotech.com/docs/5965-5978E (accessed on 03 July 2014).
9. Cai, C.H.; Regtien, P.P.L. Accurate Digital Time-of-Flight Measurement Using Self-Interference. *IEEE Trans. Instrum. Meas.* **1993**, *42*, 990–994.
10. Ji-De, H.; Chih-Kung, L.; Chau-Shioung, Y.; Wen-Jong, W.; Chih-Ting, L. High-Precision Ultrasonic Ranging System Platform Based on Peak-Detected Self-Interference Technique. *IEEE Trans. Instrum. Meas.* **2011**, *60*, 3775–3780.
11. Miller, G.L.; Boie, R.A.; Sibilia, M. Active damping of ultrasonic transducers for robotic applications. In Proceedings of the 1984 IEEE International Conference on Robotics and Automation, Atlanta, GA, USA, 13–15 March 1984; pp. 379–384.

© 2015 by the authors; licensee MDPI, Basel, Switzerland. This article is an open access article distributed under the terms and conditions of the Creative Commons Attribution license (http://creativecommons.org/licenses/by/4.0/).
